# Wild animals connect us with nature: about awe, eco-pedagogy, and nature-connectedness

**DOI:** 10.3389/fpsyg.2025.1523831

**Published:** 2025-01-29

**Authors:** Theresa S. S. Schilhab, Gertrud L. Esbensen

**Affiliations:** Danish School of Education, Aarhus University, Aarhus, Denmark

**Keywords:** wild animals, animal cognition, informal learning, connectedness with nature, eco-pedagogy, awe, Theory of Mind, embodied cognition

## Abstract

In recent years, studies have linked children’s experiences with nature to their understanding of sustainability. According to existing research, positive nature interactions and the experience of being part of nature motivate sustainable actions, a relationship described by the concept of “connectedness with nature.” Current research often refers to nature as a green (or blue) area—i.e., a place that, unlike urban areas, has characteristics that stimulate positive experiences of nature. Hence, the connection between experiences with local wild animals in nature (invertebrates such as snails and spiders, and vertebrates such as mammals and amphibians) and positive nature experiences remains unexplored. We do not yet know whether wild animals, as creatures with their own goals and worlds of experience, can stimulate children’s experience of being part of nature and ultimately lead to sustainable behaviour. However, animals are relatively easy to connect with and care for because their actions often resemble ours. This recognisability may intuitively pique children’s interest and thus initiate a budding emotional attachment to and understanding of nature. This article offers a theoretical framework for how children’s experiences of local wildlife may influence their opportunities to develop nature connectedness. The article demonstrates how observations of wild animals and their purposefulness in their natural environment potentially stimulate emotions and cognitions that are of significance to developing nature connectedness. We point to three effects, as follows: (1) the stimulation of curiosity through animals’ senses and actions, (2) insight into the diversity of nature through animals’ recognisable, yet different behaviour, and (3) the experience of how we depend on the concrete environment by proxy. We elaborate on the implications of these effects on children’s connection to nature. We also discuss the importance of adult involvement and support in the facilitation of certain feelings and cognitions in the development of children’s connectedness to nature.

## 1 Introduction

Nowadays, children have few direct experiences with nature in their immediate surroundings and spend less time outdoors than previous generations (Skar et al., 2016; Soga and Gaston, 2016). As a result, most children are unfamiliar with insects and other small creatures, neither knowing them by name nor understanding their behaviours, despite these creatures living right outside their door (Schilhab, 2021). In environmental psychology research, this trend has been referred to as “nature deficit disorder” and “extinction of experience” (Driessnack, 2009).

According to environmental education research, children’s lack of direct engagement with nature may hinder their ability to meet society’s needs for sustainable behaviour (e.g., Ernst et al., 2021). In short, a lack of nature experiences may likely cause children and young people to lose interest in sustainable habits, such as avoiding unnecessary use of harmful chemicals, opting for low-carbon transportation, or choosing eco-friendly products like sustainably produced textiles and clothing (Broom, 2017).

In this article, we hypothesise that children’s encounters with wild animals in the local environment can enhance feelings of nature-connectedness.^1^ However, scientific knowledge about this relation is scarce, as are theoretical considerations (for an exception, see Schultz, 2000). Thus, we propose and discuss how allocating time to observations of wild animals and their purposeful behaviour in natural environments potentially support both learning to care for and cognitive awareness of nature.

The focus on *direct experiences* with local wild animals stems from the fact that classroom teaching and general public education on global challenges, such as climate change and biodiversity loss, may feel too abstract to translate into sustainable actions. These issues tend to be hard to grasp unless children are placed in concrete contexts (Schilhab, 2023a,b). For example, Hicks (2018) describes how it may be necessary for geography teachers to make climate change visible in teaching—e.g., through photos of effects in the local area—so that students better grasp the issue (p. 79).^2^

Moreover, sustainable actions largely depend on factors other than school knowledge and linguistic information. This kind of knowledge is more often tied to the sharing of knowledge and practices in social communities (Broom, 2017; Esbensen et al., 2024; Hasse, 2015; Marshall and Brenneman, 2016; Schilhab and Groth, 2024). Hughes et al. (2019) emphasise the importance of social networks, values, beliefs, and attitudes, while Gifford and Nilsson (2014) identify 18 personal and social variables that affect individuals’ inclination to act sustainably. These personal factors include childhood experiences, values (closely linked to emotions, Hughes et al., 2019), self-perception, place attachment, personal goals, and chosen activities, whereas social factors encompass religion, urban–rural differences, norms, social class, proximity to environmental issues, and cultural and ethnic variations.

### 1.1 Nature connectedness

There is seemingly a correlation between the number of affective experiences with the natural environment and the desire to act in an environmentally sound manner (Hinds and Sparks, 2008). This relationship has been referred to as “connectedness to nature” (e.g., Restall and Conrad, 2015) and includes three main components [for an early definition of nature connectedness (see Schultz, 2002)]:

- Cognitive component that expresses how integrated the individual feels with nature.
- Affective component that expresses the individual’s experience of caring for nature.
- Behavioural component that expresses the individual’s experience of responsibility concerning the protection of nature.

Hence, a greater explicit understanding of nature (first component) and an elevated emotional attachment to nature (second component) constitute important factors that together contribute to developing sustainable behaviour in children (third component) (Amel et al., 2017). Importantly, if we fail to support the emotional component appropriately when communicating about sustainability, it becomes more difficult to motivate sustainable actions. This also applies, even if factors other than the emotional, as explained in the review by Gifford and Nilsson (2014), can stimulate sustainability. Kossack and Bogner (2012, p. 180) describe the challenge: “It is pointless to convey values and warnings about threats to nature when people do not feel close to nature. As long as people do not feel part of nature, they will lack motivation to engage in sustainable behaviour.”

How can we effectively stimulate children and young people’s connection to nature? One way is to facilitate both emotional attachment and the cognitive understanding that underpins how integrated the individual feels with nature.

### 1.2 Childhood experiences and connectedness to nature

The anthropologist Chawla (2007) describes how affective experiences with nature arise in childhood experiences, where natural surroundings form a backdrop for daily chores and lived life within social communities. Typically, social settings where a caregiver both verbally and physically takes the child into a community in interactions with nature will support the child’s affective experiences of nature (see also Broom, 2017; Schilhab and Esbensen, 2021; Schilhab et al., 2022).

Chawla (2007, p. 145) writes:

“When people who work to protect the environment or educate others about it are asked the reasons for their commitment, they give two answers more often than any others: special places in nature where they played as a child or hiked, camped or fished as an adolescent, and family role models who showed the value of the natural world through their own appreciative attention to it. […].”

Chawla’s findings of concrete experiences with nature and the adult role model suggest that it is both the emotional (affective) as well as the socially (e.g., the role model and/or the community) articulated (cognitive) aspect that contribute to strengthening children’s connection with nature.

### 1.3 Wild animals in nature connectedness

An important part of nature that seems easy for children to sympathise with and feel care for is wild animals (e.g., Lumber et al., 2017). Research-based interview and questionnaire studies on how zoo experiences in an educational context contribute to increased awareness of the biodiversity crisis in older children exist (Jensen, 2014; Jensen et al., 2017). However, research into the use of experiences of wild animals in their environment to develop nature connectedness in children is lacking. Studies of how interacting with wild animals (both vertebrates and invertebrates) in their environment—such as centipedes, woodlice, spiders, earthworms and slugs; as well as amphibians, snakes, and birds; and smaller mammals such as rats, mice, squirrels and hedgehogs—impact children’s emotional and cognitive development are extremely limited (Drissner et al., 2014). Modern children have far better access to (mediated) experiences with exotic and spectacular animals on other continents, such as tigers, zebras, and ostriches, than they have with animals from the local environment (Drissner et al., 2010; Snaddon et al., 2008; Strommen, 1995). Reduced contact with nature and highly specialised biology teaching also play a role in children and young people having less insight into the biological aspects of their immediate environment. According to Atran et al. (2004, p. 395), “As generations of college students learn more about microbiology and evolution, they seem to be growing less and less familiar with the plants and animals around them.”

In this article, we argue that experiences with wild animals both stimulate children to feel care for nature on an emotional level and improve their understanding of how humans relate to nature.

The article offers a theoretical framework for understanding how and why wild animals can be used to foster greater nature connectedness in preschool and primary school children. The purpose of this article is to outline a budding theory for the special role of wild animals in eco-pedagogy.

We argue that experiences with animals in their surroundings give children a direct sense of how their lives depend on their surroundings (for the importance of direct experiences, see Longbottom and Slaughter, 2016). The goal-directed behaviour of animals resembles our own because we share the same conditions. We live in the same physical and material world governed by laws and restrictions that we need to navigate. Animals’ similarities with humans make it easier for children to attribute relatable mental states to them (Burke et al., 2016; Epley et al., 2007; Varella, 2018), a practice that occurs from very early childhood (Urquiza-Haas and Kotrschal, 2015), not least through the so-called mirror neuron system that enables children to be sensitive to the actions of certain organisms (Amoruso and Urgesi, 2016; White et al., 2014). However, animals are not people, but creatures with demands that differ from ours. Hence, it is crucial that the intervention does not intrinsically create “...cognitive biases that interfere with the development of scientifically informed reasoning about natural phenomena” (Marshall and Brenneman, 2016, p. 1104). The differences are evident in their appearance and lifestyle. We unfold why this dual-sided take on animal behaviour is central to developing an understanding of why we should act responsibly toward nature.

Thus, our argument rests on three elements. First, we describe which properties of living beings enable them to mirror the circumstances of our own lives. These are the qualities that children need assistance to identify in themselves in order to understand that the maintenance of our lives depends on our surroundings. We argue that the best way to support children’s understanding of this concept is to facilitate direct contact with nature and allow pauses for them to delve and engage in observation of their surroundings. From the spotting activity, children observe the very properties that facilitate emotional reactions. We then describe how the fact that animals are distinct from people simultaneously gives children insight into how much different life there is on the planet, and how the planet provides sustenance and shelter for many species other than humans. Finally, we describe how animal behaviours, which are typically directed toward their physical surroundings, facilitate children’s own engagement with their environment, thus enhancing their experience of being in the present and enabling them to recognise their dependence on the concrete world.

The three elements discussed above involve different degrees of reflection in children. While the emotional bond can be established easily and intuitively on a non-verbal level when children experience nature in a carefree atmosphere, the explicit, more philosophical insights require focused guidance from adults. It is important to note that the budding emotional ties established when a child experiences curiosity and commitment are a prerequisite for the reflections made in the following elements. Without interest and stimulation of curiosity in the first place (first element), it becomes more difficult for children to gain insights that lead to more philosophical realisations about similarities between other beings and humans and about living creatures’ dependence on the world. In the discussion that follows, we will return to how adults play an important role in developing children’s connectedness to nature.

## 2 Three types of insights through experiences with wild animals

### 2.1 Animals perceive, respond, and behave

Everything that lives interacts with the environment in an attempt to stay alive (Godfrey-Smith, 2002; Maturana and Varela, 1987). For example, organisms must replenish lost energy to survive, since energy is required to move, reproduce, and eliminate harmful substances from the body. Organisms must also protect themselves against potential dangers, such as predators or dangerous environments that threaten their health and viability. The constant dependence on the environment in order to survive is the backbone of organisms’ ability to sense, learn, and remember (Schilhab, 2017).

For instance, when children come across woodlice in a rotten oak stump or an escargot snail wandering in the meadow, they may not immediately comprehend how these animals succeed in sustaining themselves and surviving the environment. Without adult intervention, children might notice how the snail’s antennae change shape when it is in physical contact with the outside world. They may also see, if they experience it several times, that woodlice tend to move away from light beams because they are prone to desiccation. In other words, by simply noticing their surroundings, children can observe that phenomena in the world, such as roots and light, cause snails and woodlice to behave in certain ways (Schilhab, 2021, 2024). This connection between stimulus and response characterises the living, as described by philosopher Sheets-Johnstone (1998, p. 278):

No matter what the particular world (Umwelt) in which an animal lives, it is not an unchanging world. […] Consider, for example, an earthworm, its body pressed against the earth as it crawls along, or a beetle walking along the ground. In each case, the immediate environment is tangibly inconsistent; it has topological and textural irregularities—bumps here, smoothness there, moisture here, hardness there, and so on. Both earthworm and beetle must adjust kinetically to what they find in the immediate moment.

In the above, Sheets-Johnstone refers to the Estonian biologist Uexküll’s (1864–1944) notion of Umwelt, the space of meaning in which any organism finds itself as a result of the working of its senses. Uexküll (2001) confronted the anthropocentrism of his day by demonstrating the irrevocable subjectivity and meaning-making of non-humans and humans alike (Schroer, 2021, see also theories within the field of biosemiotics, Kull and Emmeche, 2011).

Hence, the discovery of stimulus–response behaviour in other organisms does not require particular prerequisites. The discovery occurs automatically because humans are inclined to notice contingencies (Sood and Jones, 2013). Thus, children easily recognise light and darkness as external factors that are of importance to the lifeworld of the animal, especially since these are factors that children have direct experience with and attach importance to. It is worth noting that we are not arguing that children can use their perceptions and private imaginaries to understand animals in scientifically sound ways. Using the human condition as an analogy can lead to gross misunderstandings, known as anthropomorphism (Mitchell et al., 1997). However, children can use the experiences of their sensory apparatus to detect movement, colours, smells, weight, etc., in order to comprehend that animals also sense and relate to the environment and are therefore dependent on it (for a description of our stimulus dependent attentional resources see, Chun et al., 2011). In this first sense, curiosity and commitment are experienced emotionally without self-reflection. Nonetheless, Blume (2015) describes how children explore the environment to grasp what they do not understand, and thus, the opportunity to see the new arises. McBride and Brewer (2010) similarly find that more focused observations, which children may consciously decide on, often stimulate imagination and curiosity, leading to more investigations and discoveries (see also Schilhab, 2024).

### 2.2 The diversity of nature through what appears to the senses

When children discover how animals respond and behave in relation to their surroundings, they may also become aware of how diverse the world is. These experiences can lead to conceptualised insights, alongside states of interest and commitment (Malone, 2016). By experiencing the reactions and behaviours of non-human organisms, children may more easily discover parts of the world that are typically taken for granted. Adults might be so used to these conditions—e.g., that there is light and dark, that puddles vanish in the sun because water evaporates in heat, that rain increases the level of humidity—that they fade into the background. Alternatively, some conditions, such as the depressions the earthworm meets, have disappeared from our knowledge about the world due to the ubiquity of pavements (Ingold, 2021). When Sheets-Johnstone’s earthworm must deal with moisture, depressions, and the hardness of the earth, it becomes clear that the world looks different to other organisms. The behaviours of other organisms thus broaden our horizon and enlarge the world to us; hitherto insignificant aspects become increasingly important through the specific animal’s perspective. When children experience the snail’s behaviours and responses to light and dark and compare them with those of the woodlouse, they can better understand how all living creatures, including humans, have different needs. Comparisons of this nature also show that the snail, the woodlouse, and the human are just a select sample of nature’s many forms of life. Through children’s concrete contact with living creatures, the extent of earth’s complexity unfolds for them. It is vital that organisms are situated in their natural environments, that is, with affordances that support the animals’ lifecycles. In natural environments, the meaningfulness of animal behaviours and actions are more easily accessible compared to in laboratory environments (Schilhab et al., 2022). However, observing the lived lives of animals is time-consuming. Furthermore, children might need to spend time repeatedly observing animals to realise the fuller behaviour patterns of the species in question. Interpreting the behaviours of organisms in their life worlds requires insight into how shorter idiosyncratic episodes feed into an overall pattern. Are woodlice always uncomfortable with light, and are they predominantly hiding in rotting tree stumps, or could you also encounter them on a dirt road while the sun is shining?

#### 2.2.1 Insight into meaningful behaviour takes time

In comparative psychology, there has long been a division over how to methodologically understand the actions and goals of other organisms. This division serves as an example of what children can gain from repeated encounters with the same types of animals over time, rather than via single, isolated observations.

Experimental comparative psychologists often design experimental setups that test the traits of animals in laboratories; this applies, for example, to the mirror test for self-awareness (e.g., Gallup, 1970; Schilhab, 2002a, 2002b, 2004). Ethological comparative psychologists, in contrast, use more anthropologically-oriented methods, following and observing organisms over extended periods of time to form an overview of response patterns and underlying motivations behind the behaviours (e.g., Bates and Byrne, 2007; Boesch, 2021).

Primatologist De Waal (1999) explains the difference (p. 257):

“Observing animals under natural or naturalistic conditions, ethologists (behavioural zoologists) are interested in life cycles and species-typical behaviour, such as how animals defend territories, court the opposite sex, evade predators, raise their young, communicate with one another, and so on. They try to meet the animal on its own terms, comparing behavioural characteristics along phylogenetic lines. […] Behaviourists (psychologists), on the other hand, often have little interest in the animal per se. They study animals to discover general laws of behaviour and, ultimately, to understand ourselves. Their main focus is on the acquisition of stimulus-response contingencies, and the prediction and control of behaviour.”

When children repeatedly observe organisms in their natural surroundings, they can approach their discoveries in a way similar to that of ethologists. They experience and gain insight into other life forms on their terms because the investigation occurs in a relevant context, providing a more complete understanding of the relationship between life and the environment. However, it is worth noting that certain organisms may be more likely to spark children’s curiosity.

According to some studies, the ability of animals to stimulate our understanding depends on their physical resemblance to us and their emotional connection to us (Eddy et al., 1993; Rocha et al., 2016). In other studies, the key factor is the animal’s behaviour and how it fits meaningfully into a given context (Mitchell and Hamm, 1997).

Philosopher Dennett (1996) identifies three ways in which humans categorise their surroundings in his book *Kinds of Minds* (1996). Dennett calls them the physical stance, the design stance, and the intentional stance. We use the physical stance to explain and predict the existence of physical phenomena in the world. This kind of explanation includes an understanding of weight and mass. However, we use the design perspective when understanding man-made objects. This applies, for example, to the alarm clock or the kettle. When we encounter objects, we consider them based on what they were designed for, i.e., which function they perform. Living beings, on the other hand, can be described by a myriad of parameters that cannot be considered from the physical or the design stance. We therefore attempt to control this unpredictability by conceptualising their behaviours according to different kinds of “mental” phenomena, such as will, wishes, hopes, beliefs, knowledge, feelings, etc. These phenomena are also intentional, i.e., we attribute to the organism an orientation toward what it wants, aspires to, hopes to, etc., as an actor.^3^ This tendency has been confirmed in several studies. Children and adults attribute mental states to both humans and non-humans (Horowitz and Bekoff, 2007; Urquiza-Haas and Kotrschal, 2015) and like to embody and identify themselves in and with both living and narrative agents, as evidenced by fables and children’s stories (Borgi and Cirulli, 2016; De Graaf et al., 2012; White et al., 2014).

Non-human organisms in their environments influence children in ways rooted in psychological phenomena that are readily accessible to them. Animals have a special ability to spark children’s interest and empathy because their behaviours can be understood in terms of species-specific desires, needs, and even beliefs (for an illustrative example of attempts to understand the world through others’ eyes, see Yong, 2022). At the same time, animals’ distinct differences from us can trigger deeper insights, encouraging children to question why animals behave the way they do and to view them as part of the larger ecological system. Here, it is worth noting that empathising with others and understanding what they experience occur through two distinct systems (e.g., Keysers and Gazzola, 2007; Zaki and Ochsner, 2012). Emotional empathy occurs when we experience feelings as a result of observing another’s experiences. Cognitive empathy, or Theory of Mind (ToM), involves the mental process of taking another’s psychological perspective (e.g., Burke et al., 2016). Given that the emotional system is ancient, when children observe animal responses, they will likely empathise emotionally by sharing the feelings of the observed (De Waal, 2008). Such experiences could assist as drivers of the more cognitive task of recognising that animals are agents whose behaviours are formed by their perceptions of the world.

### 2.3 The animal in the present

When children observe animals in their natural environments, they also gain insight into how all living beings are embedded in their surroundings and must sense and act according to where they are. This realisation provides an opportunity to value the tangible world—specifically, the experience of being embodied and of interacting with the environment using all senses (King and Ginns, 2015; Magntorn and Helldén, 2007).

Often, that aspect of human life is at risk when, for example, we use smart technology to embed ourselves in mediated universes that support few of our senses and downplay bodily movement. We also neglect bodily engagement in the present when seated in class to learn about abstract and conceptual notions that have little to do with the body and the senses (Schilhab, 2023b).

Philosopher Rowlands (2009) story, *The Philosopher and the Wolf*, offers an example of how wild animals—in this case, a wolf—can increase our awareness of bodily interaction in the now. Rowlands acquires a Canadian wolf named Brenin, meaning “king” in Welsh, while attempting to establish a new life as a junior lecturer in the United States. Rowlands, who grew up with dogs, describes how life with Brenin is quite different from what he expected. Brenin is significantly stronger and more self-sufficient than the dogs Rowlands knew as a child. Brenin’s wild temperament manifests in more forceful actions and a stronger will. As a puppy, Brenin once dragged an armchair through the house and into the yard, damaging door frames and walls along the way. He also destroyed the house’s heating system when he became bored one day. Brenin refuses to be left alone at home, which results in him lying in the corner of the lecture hall during Rowlands’s classes, interrupting with his howling when the lectures go on too long.

Over time, Rowlands begins to notice certain behaviours in all three of his ‘canine (dog-like)’ animals (he later acquires two German Shepherds, in addition to Brenin), which stand in stark contrast to the behaviours of organisms in the order to which humans belong: primates. Rowlands describes taking daily afternoon walks with his dogs to the same beach while living in France. After their swim, they stop by the same bakery to buy croissants. Despite the routine, the three dogs remain equally excited every day, both when they realise it is time for the walk and while they are on the walk. Rowlands contrasts this with humans, who might feel less entertained by such a repetitive activity.

For Rowlands, the dogs’ enthusiasm for repetition reflects their ability to become immersed in the now and to fully engage with the physical and sensory world around them, in contrast to humans, who lose interest in the present moment to focus on plans in their imagination (for an elaborated account, see Rowlands, 2024). The dogs’ world revolves around tangible experiences—hares to chase, chairs to tame, seawater to swim in, and croissants to devour. Humans (and primates) are instead driven by future goals, perceiving life as a series of events strung together like beads on a necklace. We wake up each morning planning what needs to be done, guided by what Rowlands calls “time’s arrow,” and this suggests “a view of life’s meaning as something toward which we must aim; or as a direction in which we must travel” (2009, p. 205). In contrast, dogs are not concerned with the broader meaning, but with the moment itself, the experience of each bead (ibid, p. 206): “We see through moments, and for that reason the moment escapes us. A wolf sees the moment but cannot see through it. Time’s arrow escapes him.”

Rowlands highlights how animals—in this case, the wolf (from which dogs are evolutionarily descended)—demonstrate an immediate way of being in the world that humans have the capacity for, but do not fully cultivate.

To Rowlands, the dog, whether it be a wolf or domesticated dog, is in direct contact with the world, embracing its instincts and acknowledging the urges and sensations as a force greater than itself. Humans, by contrast, continuously look to the past or the future to seek fulfilment through their expectations of what life could be, rather than through what life actually is.

How can encounters with wild animals in local environments contribute to the type of insight Rowlands had with his dogs and wolf? When children observe creatures like woodlice, snails, ravens, or finches, they unknowingly experience the world together with the animals in the here and now. For example, the finches uncover beech nuts only after they fall from the trees above, and children may only notice the fallen nuts because the finches’ activity draws their attention to the fruit. In this way, children are physically and sensorially drawn into the present moment, as it is here that life unfolds for the creatures they observe.

Animals’ reactions to their environment emphasise that the present moment matters. Gravity exerts its force when the snail reacts to reaching the end of a branch, and the ripeness of berries determines whether the blackbird will eat them. In natural settings, children may also encounter dead animals, such as a mole or field mouse. Thus, children discover that animals once were, but are no longer, part of the present—reminders of the past and future. However, death in nature remains closely tied to the present. Carrion snails immediately detect the scent of carcasses, which become a food source for them.

Children may not consciously recognise that the animals they observe serve as reminders that, like them, we live in environments that affect our senses and bodies. This realisation requires more reflective thinking. However, it is not necessary for the experiences to have value. Simply being stimulated and having their curiosity piqued allow children to live actively in their bodies, with their senses in motion, focusing on the here and now. Over time, these experiences may contribute to children’s understanding that the present has inherent value, in part because it reveals our dependence on the surrounding world and our connection to the cycle of life.

## 3 Discussion

### 3.1 Framing nature attachment

This article has focused on how children’s experiences with wild animals impact their relationship with the world. We have elaborated on three key effects: (1) the stimulation of curiosity through recognisable stimulus–response patterns universal to humans and non-humans alike; (2) insight into the diversity of nature through animals’ recognisable, yet distinct behaviours; and (3) the recognition of our indispensable embeddedness in and dependency on the physical environment.

These three effects have been presented in a way that might suggest that when children are exposed to wild animals, the effects, especially the insights, arise spontaneously. Unfortunately, this is not the case. While local wild animals can indeed stimulate and support the development of these insights as concrete examples, psychological and anthropological research suggests that the framing of these experiences is a critical factor in determining whether the intended effects for children are achieved (Broch, 2004; King and Ginns, 2015).

For instance, children and adolescents who are unfamiliar with nature may feel out of place in such environments, and thus intimidated by what they are supposed to do or what is expected of them. In some cases, they may even be repelled by wild animals, such as spiders, snakes, or flies (e.g., Drissner et al., 2010). These early psychological reactions and response patterns can negatively influence children’s experience of animals, thereby preventing the desired outcomes from unfolding.

In practice, this means that experiences with wild animals must be framed in such a way that allow children to see animals as beings that, like us, are dependent on their surroundings (for such informal learning situations, see Hasse, 2015). Children need to be socialised into recognising organisms and understanding how their actions and intentional behaviours reflect a meaningful navigation of the world. This framing requires guidance on two levels.

The first level involves creating an environment in which children feel comfortable with the act of observation itself. This enables them to become accustomed to and familiar with the conditions under which animal observations take place. For example, this includes learning to dress appropriately for being outdoors, becoming comfortable with potentially getting wet socks in rubber boots, and accepting that food and drink might need to be shared with ants and wasps. Most significantly, it involves teaching them how to interact with living creatures in an ethically responsible manner.

The second level focuses on guiding children to identify what is interesting, to endure the fact that observations are sometimes tedious, and to understand that noticing something remarkable is not necessarily reflected by saliency, but just as often requires patience and time. Moreover, appropriate guidance encompasses learning why and when such experiences can be enjoyable, especially when shared with others. The idea that meaningful experiences take time has become more challenging in an era where immediate feedback and stimulation are readily accessible through smart technology (Greenfield, 2015).

Both levels involve types of learning that can be categorised as informal learning, which occurs spontaneously and is often driven by the individual’s own motivation: “I do not want to be cold,” “The ant runs faster than I expected,” “It feels nice to share experiences with others,” etc. Additionally, both levels depend on the framing provided by adults as gatekeepers who open up opportunities by creating the necessary conditions for children to experience these events in the right emotional contexts and to acquire the tools needed to navigate informal learning episodes—often through reflective and personally engaging conversations (Balling et al., 2022; Beck, 2024; Reider et al., 2023; Schilhab and Esbensen, 2019; Schilhab et al., 2024).

The significance of adult beliefs and values in shaping children’s ability to connect with nature is highlighted in an Australian study that examined parents’ and educators’ perspectives on children’s play in natural environments (Dankiw et al., 2023). The study revealed that many parents are concerned about their children getting dirty or ruining their clothes while in nature. Parents also fear that their children might get injured because play in nature tends to involve more risks than playing on controlled surfaces like playgrounds. These concerns are often passed on to educators, who must account for worried parents while ensuring that the overall structure of the children’s day remains smooth. The study emphasises that social environments are crucial in determining whether children are physically allowed access to nature and thus whether they can build experiences with it. If the social door remains closed, nature is inaccessible.

Social environments also manifest through role models, as described by Chawla (2007) in the context of nature connectedness. When children interact with adults who point out what is exciting and fun in nature, they are invited to participate fully in these experiences and can imitate the adults’ enthusiasm (see Hasse, 2015). This guidance is particularly important because it is always adults who unfold children’s worlds. If you do not give attention to the woodlouse scurrying past your foot, how can your child or student be expected to maintain interest? If you do not dress warmly and appropriately for the rain, how will your child or student know how to do so? And if you do not talk to your child or student about how we are dependent on the oxygen in the air and need food and shelter just like all other organisms, how will they discover that for themselves?

Chawla (2007) describes the formative significance of joint attention episodes in the lives of environmentalists (p. 158) and concludes:

“…that significant adults gave attention to the environment in four ways: by expressing care for the land as a limited resource essential for family identity and well-being; by disapproving of destructive practices; by sharing pleasure at being out in nature; and through their own fascination with details of the earth, sky, and living things.”

Social contexts also impact expectations of how to be a good caregiver (or educator). If the societal expectation is that children’s clothes remain clean and neat, it is more difficult for parents to ignore when their children’s clothing is dirty, even if doing so helps their children become more connected to nature (e.g., Schilhab and Esbensen, 2019).

### 3.2 Framing the time expenditure

Framing also involves the consideration of time spent on observation activities. Accessing the behaviour of organisms and discovering how life meaningfully unfolds does not materialise instantaneously. Such endeavours require a certain amount of perseverance to succeed. Therefore, children must be allowed repeated experiences to obtain lasting insights. Imagine, for example, how chickens communicate. You must be in the right spot at the right time to perceive the subtleties of their interactions. Only through several experiences with hens’ varied forms of communication in distinct contexts—e.g., food, mates, enemies, conspecifics, threats, unknown situations, whether they have chicks, are injured, are young birds, etc.—will a coherent understanding about when and under which circumstances the animals show certain types of behaviours emerge.

We are not suggesting that children should train as ethologists, but rather that children benefit from learning to pause and to follow thought patterns ignited by long-term observations. Similarly, a connection with nature is developed through sensations, values, and feelings established and shaped by many episodes in varied situations. This typically requires commitment and substantial time to develop.

### 3.3 Wildness and awe

This article has focused on wild animals, but one might ask whether similar experiences could be gained with domesticated animals. For instance, Rowlands describes how both his German Shepherds and his wolf seamlessly interact with their surroundings. Similarly, pet owners seem to have easy access to their animals’ meaningful lives, so why must children specifically engage with wild animals? In fact, it has already been suggested that children who have cared for pets develop a deeper conceptual understanding of these animals’ biology and their emotions (Prokop et al., 2008; Rocha et al., 2016).

However, the three aspects highlighted in this article focus on fostering children’s awareness of human dependence on the planet, in order to contribute to their sense of nature connectedness. Thus, we have proposed how engaging with wild animals can cultivate an understanding of life’s autonomous existence (first argument), life’s diversity (second argument), and life’s dependence on what is directly experienced in the now, to endorse an appreciation of the tangible (third argument).

Although the first and third relationships apply to domesticated animals, we argue that wild animals have traits that invoke a particular attitude in us. Pets, like all life, are self-sustaining entities. However, it is doubtful that this feature among pets stands out as significant to children. More likely, children learn that pets must submit to humans and are deeply dependent on humans to be their protectors (e.g., Borgi and Cirulli, 2013). This interpretation is reinforced by the fact that we encourage calm and gentle behaviour toward pets through treats and care. Thus, the pet benefits from behaving in a domesticated manner.

The attitude that wild animals evoke in us can best be described as a sense of awe (Keltner, 2023; Keltner and Haidt, 2003). Rowlands touches on this feeling as well. In an attempt to channel his wolf Brenin’s immense energy, Rowlands begins running with him. He discovers that Brenin’s running skill is incomparable to both his own and his domesticated dogs’ running skills. Although domesticated dogs are taxonomically descended from wolf ancestors, domestication has reduced some of their wildness, as Rowlands notes in his interactions with his animals.

When Brenin runs, he merges into his running abilities. He strides over the fields in graceful, complete movements, as if Brenin, in the act of running, becomes the very embodiment of his abilities. His seamless running style makes him a perfect match with the terrain, leaving Rowlands with a sense of taking part in something far greater than himself. Meanwhile, Rowlands struggles with injuries and experiences his arms and head flailing awkwardly, even though the running improves his fitness (Rowlands, 2013).

Despite having been raised by Rowlands and naturally undergoing socialisation, Brenin is still made for life in a world untouched by human influence. Rowlands sees this in Brenin’s raw power, when the wolf tears apart the home in impatience during Rowlands’ lectures and when the wolf graceful runs.

In short, wild animals can help us experience a sense of our own insignificance. That is, we move away from an anthropocentric perspective of ourselves and instead recognise the planet and the opportunities it has provided us as humans. This is not a state we expect children to fully understand and articulate, upon encountering wild animals. However, the three effects discussed above offer the potential for this realisation to take root, fostering a connection to nature through its grandeur.

The philosopher Nietzsche offers a thought-provoking interpretation of this feeling, which can help us view ourselves from the outside—just as wild animals can help children do by serving as analogies (Nietzsche, 2006, p. 114):

Once upon a time, in some out of the way corner of that universe which is dispersed into numberless twinkling solar systems, there was a star upon which clever beasts invented knowing. That was the most arrogant and mendacious minute of “world history,” but nevertheless, it was only a minute. After nature had drawn a few breaths, the star cooled and congealed, and the clever beasts had to die. – One might invent such a fable, and yet he still would not have adequately illustrated how miserable, how shadowy and transient, how aimless and arbitrary the human intellect looks within nature. There were eternities during which it did not exist. And when it is all over with the human intellect, nothing will have happened. For this intellect has no additional mission which would lead it beyond human life. Rather, it is human, and only its possessor and begetter takes it so solemnly—as though the world’s axis turned within it.

This attitude involves an ecocentrism that acknowledges that “we are part of a ‘more-than-human community,’ a community that is not only physical but also ethical and extends to all of nature” (Gjerris, 2019, p. 56 on Abram, 1996).

We do not achieve this insight to the same extent through interactions with pets, which are—through domestication and daily training—shaped in our own image (Borgi and Cirulli, 2016).

Fascination with biological diversity, awe, and surprise at the complexity of the world do not, as one might fear, stand in opposition to scientific knowledge. The emotion-laden sense of awe, according to Sheets-Johnstone (2023), was keenly felt by Charles Darwin, the world’s most renowned biologist, in his scientific endeavours. Focusing on wild animals and their embeddedness in the world is a valid biological approach that may even help us better care for the planet. However, we do not need to visit the Galapagos Islands to experience this. Our local environments are more than sufficient.

An anthropological interview study of American-European and Native American (Menominee) children, aged five to seven, reveals the profound influence of our cultural upbringing on what we value (Unsworth et al., 2012). The researchers found that Menominee children were more likely to mention ecological relationships and expressed a greater connection to nature, as well as a tendency to imitate wild animals in their conversations, showing signs of empathising with their world. The researchers concluded as follows (ibid, p. 26):

This work may have implications for the understanding of orientations toward nature, to the extent that ecological reasoning reflects an appreciation of the environment as a system of dependencies and animal mimicry reflects first person perspective-taking of animals. More research is needed to explore these possibilities and to examine the kind of cultural input that might support children’s learning of cultural orientations toward nature.

The study’s analysis of the cultural significance emphasises that more than an individual’s desire to seek nature is required to truly stimulate a connection with it—echoing Chawla’s (2007) and Dankiw et al.’s (2023) findings.

We must socialise future generations with an agenda that highlights knowledge of and awe for the planet we inhabit through everyday activities where conceptual understanding and informal knowledge go hand in hand (see, for example, Schilhab, 2023a, 2023b). Previous studies suggest that interacting with local wildlife is a sensible place to start.

### 3.4 Human nature

What is the importance of stimulating a connection to nature? In this article, we have only briefly touched upon the fact that children benefit from experiences with nature, regardless of how these experiences may influence their ability to act sustainably later in life (Schilhab et al., 2018; Stevenson et al., 2018, 2019). We briefly addressed this aspect in the third argument, supporting the experience of the present as a springboard to discover the possibilities the planet offers. To be clear, children, like adults, also need to experience their bodies and senses in the way that only uneven terrain, wind, and weather can stimulate them, without those experiences having to be a means to anything else.

We should clarify that implicit to the arguments, is, that informal and formal learning processes are constituted by whole-body activities in line with the Embodied Cognition approach now gaining foothold in the cognitive sciences (e.g., Hesslow, 2012; Rowlands, 2010; Schilhab, 2011, 2021; Schilhab T, 2015; Shapiro and Stolz, 2019).

People (and organisms for that matter) are always immersed in a physical and social environment, and engaged in activities that often involve various objects, for examples books, mobile phones, binoculars or bicycles, their learning is dynamic, enactive and depending on experiences (Barsalou, 2009; Hillesund et al., 2022; Schilhab and Groth, 2024).

As succinctly phrased by Glenberg (2015, p. 165):

Few still believe humans are unrelated to the rest of the animal kingdom, and soon few will believe human thinking is computer-like. Instead, as with all animals, our thoughts are based on bodily experiences, and our thoughts and behaviours are controlled by bodily and neural systems of perception, action, and emotion interacting with the physical and social environments. We are embodied; nothing more. Embodied cognition is about cognition formatted in sensorimotor experience, and sensorimotor systems make those thoughts dynamic. Even processes that seem abstract, such as language comprehension and goal understanding are embodied. Thus, embodied cognition is not limited to one type of thought or another: It is cognition.

These “biological roots of human understanding” (see Maturana and Varela, 1987) warrant why wild animals are intrinsically interesting to us and why we must cultivate methods that aim at reconnecting us and future generations with nature (Bekoff and Bexell, 2010; Schilhab T. S, 2015).

Children also benefit from experiencing awe and being part of something bigger, even if the experience does not directly lead them to behave sustainably later in life. Research shows that the experience of being part of a larger whole is linked to increased satisfaction with life (Bai et al., 2021). Being able to both appreciate the now and to feel awe concern what children immediately gain from experiences with wild animals and not what society needs them to learn. These are important effects in themselves that we have not addressed in the present article. Instead, we have emphasised how nature experiences can contribute to children becoming better at preserving the world. With that perspective, however, one might lose sight of the importance of appreciating the concrete, as described in our third argument.

However, is not staying in the present contrary to human nature, which is also distinguished by our ability to conceptualise the past and the future? Isn’t a specifically human attribute our ability to plan and let ourselves be guided by the goals we define? And is it not also that very inclination—to plan—that will support and guide us, when we need to acquire new sustainable social habits: after all, we must be able to visualise something that does not yet exist in order to navigate our way out of current global crises.

Nevertheless, we argue that humans can also be stimulated by nature (Ulrich et al., 1991). However, this ability risks being drowned out by our self-created values, so humans do not see that we, like all other living creatures, are part of a larger whole. This is where wild animals and their relationships with their environment can help humans find our way back to the common abilities that we share with them. We are all nature. We are all equipped with abilities to navigate the world. When we experience the navigation of other organisms in the world, when we see them handling environments meaningfully, we are reminded that the world exists and that we are a part of it.

### 3.5 Practical implications

What are the implications of the threefold argument for caregivers, educators, and society as a whole?

First, as stated by Chawla, parents and caregivers are significant role models and curators of safe nature spaces for children. They are the primary gate-and time keepers allowing children the luxury of dwelling and immersing themselves in the dynamics of the natural world. However, contemporary adults feel increasingly incompetent about their knowledge of nature. Hence, they are distinct from the significant adults who, following Chawla, gave attention to the environment in an informed way. This perceived lack of knowledge can lead caregivers to avoid nature-based family-friendly activities.

Here, surprisingly, smart-technological solutions in the form of nature apps that can identify by photo recognition or invite play actions like geocaching may create the “scaffold” necessary for adults to feel confident in the situation (Balling et al., 2022; Esbensen et al., 2024; Esbensen and Schilhab, 2024; Jepson and Ladle, 2015; Schilhab and Esbensen, 2021).

It is worth noting that the sought-after nature experiences are not reserved for exotic and rare landscapes. Parents and caregivers should not constantly orchestrate sophisticated sceneries to support the sense of nature connectedness. It is quite the opposite. Parents and caregivers should create room for observations of the most insignificant and common organisms in the immediate environment. Together, the child–parent dyad could explore the humble creatures which live in our vicinity and thus inspire the child to discover the mystery and otherness all around.

Likewise, school administrations must support teachers financially and logistically, so they implement excursions and longer stays in nature as part of their teaching routines. Teachers should increasingly take advantage of school grounds and school gardens to stimulate an understanding of the insects and smaller mammals in students’ immediate environments.

Also, teachers must embrace the fact that the teaching technique is radically transformed in nature. As discussed in Schilhab (2021, p. 14):

…teaching in a natural environment typically follows a more open course because the outdoor space varies in terms of its organic environment. If organisms are not in the pre-planned location, this might obstruct the teaching (Glaab and Heyne, 2020; Schilhab and Lindvall, 2017).

Also, the teacher must learn to grasp spontaneous learning opportunities without feeling the pressure from their supposed obligation to strengthen students’ abilities when tested (Carrier et al., 2013).

Last, society must develop a new vocabulary for how humans learn. This vocabulary builds on the “biological roots of human understanding” and embodied cognition, rendering our connection to nature and wild animals undeniable.

## Data Availability

The original contributions presented in the study are included in the article/supplementary material, further inquiries can be directed to the corresponding author.

## Footnotes

^1^This article draws on research published in Schilhab et al., 2022; Schilhab and Esbensen, 2019, 2024.

^2^We thank one of the anonymous reviewers for highlighting that in many countries, teaching efforts are not necessarily up to the teacher’s individual beliefs or decisions (for elaborated discussions about barriers set by school policies, see also Schilhab, 2021).

^3^It is still highly controversial, whether animals experience consciousness in senses similar to humans (e.g., Griffin, 2001). We do not intend to discuss this issue here. However, it is worth noting that opposed to Dennett’s point of view that animals behave ‘as-if’ they experience consciousness, we take the presented claims about animal stimulus–response patterns, self-sustaining organisation, and relatable dependence on their surroundings to point also to similarities in phenomenal experiences between humans and non-human animals (Andrews, 2020; Schilhab, 2015a). In other words, we assume that for a creature to be phenomenally conscious, there is something it is like to be that creature (see Rowlands, 2009, p. 50).
